# Antibacterial and Antioxidant Properties of the Leaves and Stem Essential Oils of* Jatropha gossypifolia* L.

**DOI:** 10.1155/2016/9392716

**Published:** 2016-10-24

**Authors:** Sunday O. Okoh, Benson C. Iweriebor, Omobola O. Okoh, Uchechukwu U. Nwodo, Anthony I. Okoh

**Affiliations:** ^1^SAMRC Microbial Water Quality Monitoring Centre, University of Fort Hare, Alice 5700, South Africa; ^2^Applied and Environmental Microbiology Research Group (AEMREG), Department of Biochemistry and Microbiology, University of Fort Hare, Alice 5700, South Africa; ^3^Department of Pure and Applied Chemistry, University of Fort Hare, Alice 5700, South Africa

## Abstract

Antibacterial and antioxidant properties of the leaves and stem essential oils (EOs) of* Jatropha gossypifolia* and their efficacies against infectious and oxidative stress diseases were studied* in vitro*. The EOs obtained using Clevenger modified apparatus were characterized by high resolution GC-MS, while their antioxidant and antibacterial properties were examined by spectrophotometric and agar diffusion techniques, respectively. The EOs exhibited strong antibacterial activity against* Escherichia coli*,* Enterococcus faecium, *and* Staphylococcus aureus*. The stem essential oil (SEO) was more active than the leaf essential oil (LEO) against test bacteria with minimum inhibition concentration (MIC) ranging from 0.025 to 0.05 mg/mL and the LEO from 0.05 to 0.10 mg/mL. The SEO was bactericidal at 0.025 and 0.05 mg/mL against* S*.* aureus* and* E*.* faecium*, respectively, and the LEO was bacteriostatic against the three bacteria at 0.05 and 0.10 mg/mL. The SEO IC_50_ (0.07 mg/mL) showed that the antiradical strength was superior to LEO (0.32 mg/mL) and *β*-carotene (1.62 mg/mL) in scavenging 2, 2-diphenyl-1-picrylhydrazyl radicals (DPPH^•^). The oils effectively reduced three other oxidants to neutral molecules in concentration dependent manner. Findings from this study suggest that, apart from the traditional uses of the plant extracts, the EOs have strong bioactive compounds with noteworthy antibacterial and antiradical properties and may be good candidates in the search for lead compounds for the synthesis of novel potent antibiotics.

## 1. Introduction

The rising challenge of resistance of bacteria to many antibiotics has elicited the need for the development of novel therapies with little or no side effect, to effectively manage many infectious diseases [1]. Noteworthy phytochemicals results in recent years suggest essential oil as better option due its superior properties and hence may stand in place of antibiotics to overcome known infective bacteria species as well as yeasts and filamentous fungi [2–4]. Constituents of essential oil are numerous, complex, and known to possess strong antibacterial property, especially polyphenol, aliphatic and cyclic terpenes, oxygenated terpenes, and phenylpropenes [5–7]. Essential oils have been shown to passively diffuse cell membrane of bacteria owing to their permeability properties across biological lipid barriers [4, 6]. This membrane interaction can lead to membrane instability consequently resulting in the leakage of the bacterial important intracellular components and ultimately cell death occurs [6]. Cell wall, cell membrane, intracellular proteins, nucleic acids, enzymes, and few others are vital target sites for drug design and some essential oil compounds have these specialized parts of the cell as important receptor targets [8].

Enzymatic antioxidant defense systems comprising superoxide dismutase (SOD), catalase (CAT), glutathione peroxidase (G-Px), and other endogenous antioxidant molecules, notably glutathione (GSH), do scavenge oxygen derived free radicals produced in physiological and pathological processes. However, the inhibition of such reactive oxygen derived species such as superoxide (O_2_^•^), nitric oxide (NO^•^), hydroxyl (HO^•^), and lipid peroxyl (LP^•^) generated from body metabolic activities as well as environmentally induced radicals overwhelms the bodies natural defense antioxidants [9–12]. Furthermore, studies have shown that there is decline in viability and potency of the human's antioxidants as individual ages [13, 14]. Man has used spices, decoctions, fruits, vegetables, and infusions which are now acknowledged as containing potent secondary metabolites against diseases before the appearance of the written word. In the last two decades, several studies have shown secondary metabolites including phenolics, flavonoids, alkaloids, and essential oil compounds as potent antioxidants [15–17]. Essential oil could function as a credible option to synthetic antibiotics due its ability to penetrate microorganism cell membrane resulting in inhibition of microorganism growth as well as capacity to quench free radicals [6, 17]. In addition, there are growing concerns on the use of nonnatural preservatives by consumers and food processing industries owing to their reported adverse effects. Studies by Wang et al. [17] on some essential oil constituents revealed that, unlike the synthetic free radical scavengers, the byproducts of natural antioxidants are presumably safe and may be preferred in reducing the total oxidative stress. Plant essential oil components, including limonene, linalool, menthol, and caryophyllene, reported to possess such significant bioactive properties have been registered by European Commission as flavors for use in food products [9, 18].

*Jatropha gossypifolia* (Euphorbiaceae) is a traditional medicinal shrub plant applied for management of skin diseases, diabetes, and cancers [19]. In Nigeria, fresh leaf aqueous extract is utilized in folk medicine for healing of mouth cancer and to terminate skin and nose bleeding while the stem is served as brush for healthy tooth [20, 21]. In India leaves are used for prevention and treatment of variety of diseases including dysentery, eczema, diarrhea, and itches [22]. Decoction of* J. gossypifolia* in Trinidad and Tobago was found potent for treating wound, reducing pain, and treating snatch sores [23]. Phytochemicals analyses have shown that different parts of* J. gossypifolia* contain phenolics, flavonoids, and alkaloid compounds [22, 23]. Aboaba et al. [24] reported phytol, germacrene, and linalool as some of the leaf volatile oil constituents of* J*.* gossypifolia*.

There has been dismayed rise of bacterial resistance to currently available antibiotics; this has motivated a search for alternative sources of antimicrobial agents which are believed to be found abundantly in plants. There is however dearth of information on comparative evaluation of the antimicrobial and antioxidant properties as well as the bioactive volatile constituents of the stem and leaf essential oil of* J. gossypifolia*; hence this current study aimed to evaluate the antibacterial and antioxidant properties of the leaves and stem essential oils of* Jatropha gossypifolia*.

## 2. Materials and Methods

### 2.1. Analytical Reagents

The chemicals and reagents used included the following: Mueller-Hinton agar from Oxford Ltd. (Hampshire, England), dimethyl sulfoxide (DMSO), and methanol from Fluka Chemicals (Buchs, Switzerland). 2,2′-Azino-bis(3-ethylbenzothiazoline-6-sulfonic acid) diammonium salt (ABTS), butylated hydroxyl toluene (BHT), and 2, 2-diphenyl-1-picrylhydrazyl (DPPH) were bought from Sigma-Aldrich (St Louis, USA). All chemicals and reagents used were of analytical grade.

### 2.2. Plant Material

*J. gossypifolia* was obtained from Forest Research Institute of Nigeria (FRIN), Ibadan, Oyo State, Southwest Nigeria. A plant taxonomist authenticated the plant and samples were kept in the Lagos University herbarium (LUH) with voucher specimens numbers LUH2009 and LUH2011 for the leaf and stem, respectively. The leaves were sufficiently air-dried in 5 days at the ambient room temperature, while the stem was cut into smaller pieces and air-dried in 7 days. They were pulverized and essential oil was extracted for 3 h from each (200 g) using modified Clevenger-type apparatus [25]. The hydrodistillation experiment was carried out twice for the leaf and stem separately to obtain enough oil for bioactivity assays. The extracted essential oils were dried over anhydrous sodium sulphate, dispensed into tinted vials, and stored at 4°C. The yield of each essential oil was computed in w/w% (per gram) of individual hydrodistilled plant sample.

### 2.3. Characterization of Essential Oils by Gas Chromatography-Mass Spectrometry (GC/MS)

We employed GC/MS to analyze and identify the essential oil constituents. The GC-MS conditions were programmed as previously described [26], in which the mass spectrometer (Hewlett-Packed HP 5973) interfaced with an HP 6890 gas chromatograph. Conditions of the temperature and column were as follows: equilibration time 3 min, ramp 4°C/min, initial temperature 70°C, and final temperature 240°C; inlet: splitless, initial temperature 220°C, pressure 8.27 psi, purge flow 30 mL/min, purge time 0.20 min, and helium gas; column: capillary, 30 m × 0.25 mm, internal diameter 0.25 *μ*m, film thickness 0.7 mL/min, and average velocity 32 cm/sec; MS: EI method at 70 eV. Subsequently, identity of each constituent was ascertained by using agreement of its mass spectra data (MSD) of Wiley 275, New York reference computer library. In addition, matching the retention index (RI) of each compound with those in literature was also employed in identifying the compound. The peak areas were used to obtain total percentage composition of oil.

### 2.4. Antibacterial Activity

#### 2.4.1. Bacteria Suspensions Test

Antibacterial activities of the oils were tested against three bacterial strains comprising two Gram-positive bacteria reference strains,* S. aureus* (NCINB 50080) and* E. faecium* (ATCC19434), and* E. coli* O157, as a Gram-negative bacterium (ATCC 700728), following the guideline recommended by CLSI (2014). These reference strains were grown in Muller Hinton broth at 37°C for 24 h. Minimum inhibitory concentration (MIC) as well as minimum bactericidal concentration (MBC) potentials was performed on Muller Hinton agar plates at 37°C for 24 h. Ciprofloxacin was applied as reference standard (RS) or positive control.

#### 2.4.2. MIC and MBC Evaluation

The microdilution technique was carried out to evaluate the MICs. 800, 875, 900, 950, 975, and 987.5 *μ*L of Mueller-Hinton broth (MHB) were added to each Eppendorf tube. Five hundred milligrams of both SEO and LEO stocks after evaporation of n-hexane was separately dissolved in DMSO (500 *μ*L) and each solution was vortexed. Thereafter, aliquots of 200 *μ*L, 125 *μ*L, 100 *μ*L, 50 *μ*L, 25 *μ*L, and 12.5 *μ*L were added, respectively, to each tube containing MHB to bring the final volume in each to 1 mL and the mixtures were properly vortexed. The inoculum suspension (20 *μ*L) of each tested bacterium (0.5 McFarland, ~1 × 10^8^ cfu/mL) was added subsequently and vortexed to permit adequate mixing of the essential oil and broth. Ciprofloxacin and DMSO served as the positive and negative controls, respectively. The experiments above were performed in duplicate and incubated at 37°C for 24 h. Tubes with lowest concentration without visible growth were reported as the MIC. MBC was tested by streaking out all wells without visible growth in the MIC technique above onto fresh nutrient agar plates and the culture was incubated for 24 h at 37°C. The lowest concentration of extracts that did not yield any growth on the solid medium after the incubation period was recorded as minimum bactericidal concentration (MBC).

### 2.5. Antioxidant Property

DPPH, ABTS, nitric oxide, and lipid peroxyl radicals inhibiting tests were performed to determine the antiradical property of the two essential oils.

#### 2.5.1. DPPH Assay

The DPPH test was carried out as described by Liyana-Pathirana and Shahidi [27]. Briefly in DMSO a solution of DPPH (2.7 *μ*M) was made; afterwards 1 mL of it was vortexed with 1 mL of the essential oil dissolved in DMSO which has 0.025–0.50 mg/mL of the oil as well as the reference standard (RS). Then, the reaction mixture was vortexed and incubated in the dark for 30 min at ambient temperature. The absorbance of the reaction mixture was then read at 517 nm against a reference blank containing DMSO. The assay was carried in triplicate and DMSO was used as blank. Essential oil's potency to reduce DPPH^•^ to neutral molecule was computed as inhibitory percentage using the expression:

(1) %  inhibition  of  DPPH•  by  EO  or  RS=Abscontrol−AbssampleAbscontrol×100,

where Abs_control_ is the absorbance of the DPPH radical + DMSO and Abs_sample_ is the absorbance of DPPH radical + essential oil or reference standard.

The IC_50_, that is, concentration of the essential oil or reference standard (positive control) required to reduce 50% of the DPPH^•^, was obtained from the standard curve produced with varying concentrations versus inhibitions and results compared to that of reference standard.

#### 2.5.2. ABTS Radical Scavenging Assay

The ABTS radical scavenging assay procedure was carried out following the method of Re et al. [28] with some modification as described by Witayapan et al. [3] by mixing 1 : 1 volumes of ABTS 7.0 mM and 4.9 mM potassium persulfate solution. The mixed solution was kept at room temperature for 12 h in a dark chamber. The ABTS radical cation (ABT^•+^) was then diluted with DMSO to equilibrate its absorbance to 0.705 (±0.001) at 734 nm. To carry out the assay, 1000 *μ*L of 0.025–0.50 mg/mL solutions of the test samples (SEO and LEO) in DMSO was mixed with 1000 *μ*L ABT^•+^ solution, bringing final volume of each mixture to 2 mL. The mixture was allowed to react for 7 min. The absorbance at 760 nm was measured spectrophotometrically and the assay was carried out in triplicate. The radical scavenging activity of the EO or RC was expressed in terms of percentage (%) inhibition of ABTS^•+^ using expression in (1) described in Section 2.5.1.

#### 2.5.3. Inhibition of Lipid Peroxidation by TBARS Assay

The inhibition of lipid peroxidation formation by the essential oils was measured using an adaptation of the method described by Badmus et al. [29] with egg yolk as lipid rich media. To a 10% egg yolk homogenate (0.5 mL) was added 0.1 mL of the test samples (in DMSO) at varying concentrations (0.025–0.50 mg/mL) and the reaction mixture made up to 1 mL. The lipid peroxidation was induced by adding 0.05 mL of 0.07 M FeSO_4_ and the mixture was then incubated for 30 min. Then, 1.5 mL of 10% acetic acid (pH 3.50) and 1.5 mL of 0.08% 2-thiobarbituric acid (in 1.1% sodium dodecyl sulphate and 20% trichloroacetic acid) were added and the mixture was vortexed and then heated at 65°C for one hour. Upon cooling, 0.5 mL of n-butanol was added to reaction mixture and centrifuged for 10 min at 3000 rpm. The upper organic layer was then aspirated and the absorbance read at 532 nm. The percentage inhibition of lipid peroxidation was calculated using the expression in equation as described in Section 2.5.1.

#### 2.5.4. Nitric Oxide Radical Inhibition Assay

The nitric oxide radical scavenging activities of the essential oils were carried out according to the modified method described by Makhija et al. [30]. The compound sodium nitroprusside is known to decompose in aqueous solution at physiological pH (7.2) producing nitric oxide radicals (NO^•^). Under aerobic conditions, nitric oxide radicals react with oxygen to produce stable products (nitrate and nitrite) which can be measured using Griess reagent [31]. To 1 mL of sodium nitroprusside solution (10 mM) was added 1 mL of the essential oil at varying concentrations (0.025–0.5 mg/mL) and the mixture was then incubated at ambient temperature for 110 min. After incubation, 1 mL of the reacting mixture was added to Griess reagent (1%, sulphanilamide, 1% N-naphthyl-ethylenediamine hydrochloride in 2% o-phosphoric acid). The absorbance of the color developed was then measured at 546 nm against the reagent blank. The assay was carried out in triplicate and percentage inhibition was calculated using the expression in (1).

### 2.6. Statistical Analysis

The results are expressed as the means ± SD for triplicate assays. Linear regression analysis was used to calculate IC_50_ values while Pearson's correlation analysis and* t*-test were used to test for significance between concentration and percentage inhibition using SPSS 15.0 for windows (SPSS Inc.).

## 3. Results and Discussion

### 3.1. Composition of the Essential Oils Extracted

The gas chromatography-mass spectrometry qualitative and quantitative analyses of the essential oils of* J. gossypifolia* in our previous report [32] and the present study revealed that constituents of the leaf essential oil (LEO) are predominantly alcohols including phytol (33.40%) and linalool (9.81%) presented in Table 1. Out of the 15 constituents identified in LEO accounting for 98.70% of the total oil content, four were among the* J. gossypifolia* leaf oil components in Aboaba et al. [24]. In addition to phytol (18.05%) and other terpenoids constituents, more monoterpenes and sesquiterpenes including limonene (12.40%), germacrene D (12.30%), *α*-copaene (12.20%), *α*-terpinene (10.61%), and *α*-aromadendrene (10.48%) were identified as major compounds in the stem essential oil (SEO) than in the LEO in this study. Lanosterol, humulene, 2, 6-di-butyl-p-cresol, heptadecanoic acid, and linoleic acid have also been reported as constituents of LEO of* J. gossypifolia* [33, 34] but they were however not found in this study. The discrepancy in the composition of* J. gossypifolia* essential oil grown in different regions in Nigeria and elsewhere may be due to differences in factors, such as climatic, seasonal, and geographical conditions, age of plant, humidity of the harvested plant material, extraction technique, and the existence of chemotype [35].

### 3.2. Antibacterial Activity of the Essential Oils

The essential oils extracted from the leaves and stem of* J. gossypifolia* strongly exhibited inhibitory activity against the 3 bacteria strains (*Escherichia coli*,* Enterococcus faecium*, and* Staphylococcus aureus*) investigated. The stem essential oil (SEO) MIC values of 0.025 ± 0.01, 0.05 ± 0.00, and 0.05 ± 0.00 mg/mL showed that it is more active than the leaf essential oil (LEO) with MIC values of 0.05 ± 0.00, 0.10 ± 0.01, and 0.10 ± 0.01 mg/mL against* E. faecium*,* S. aureus*, and* E. coli, *respectively (Table 2). Similarly 0.025 mg/mL of SEO was able to kill (bactericidal)* E. faecium*, while it requires twice the dose (0.05 mg/mL) to exhibit bactericidal activity against* S. aureus*. Unlike the two Gram-positive bacteria tested, the oils were less active against Gram-negative bacterium (*E. coli*). However, at 0.10 mg/mL the SEO was bactericidal against* E. coli* while the LEO was bacteriostatic at the same concentration (Table 3). The differences in antibacterial property could be due to net repulsion of the two outer complex membranes' structure (a two-lipid bilayer) in Gram-negative bacterial cell wall which is absent in Gram-positive bacteria [36]. These layers constitute physical barriers between microorganism and the environment, preventing interactions of the bacterial cell with harmful substances. A Gram-positive bacterium has only one relatively thick permeable membrane, rendering it more susceptible to interactions with the environment [37]. The effects of the stem and leaves oils of* J. gossypifolia* against the bacteria also differed; the variation observed in the chemicals profiles of two oils may possibly account for their varied bioactivity [38, 39] in the present study.

### 3.3. Antioxidant Activity of the Essential Oils

**Figure 1:**
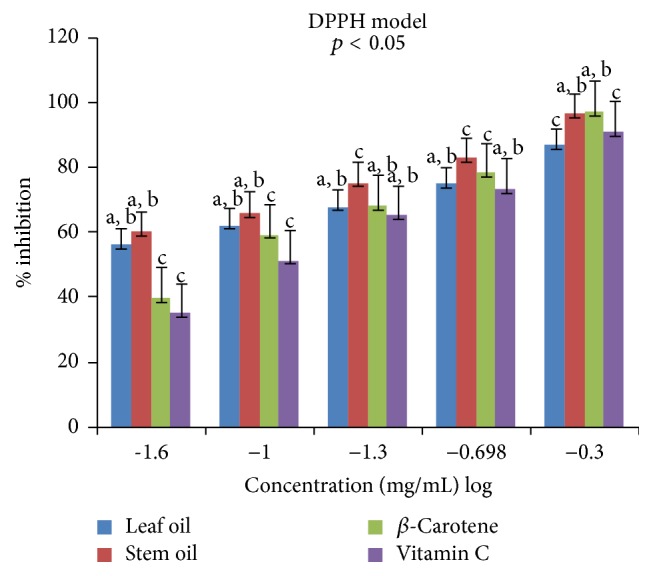
Antiradical effects of leaf and stem oils of* J. gossypifolia* and reference standard on DPPH radicals: a, b, not significantly different; c, significantly different (*p* < 0.05).

**Figure 2 fig2:**
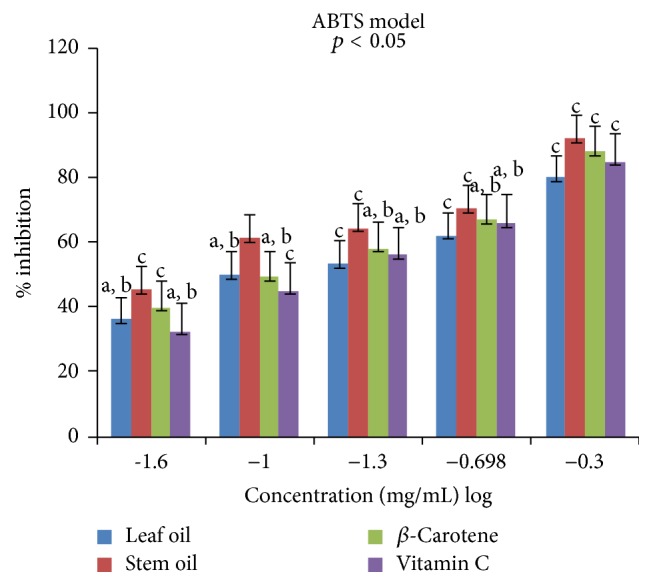
Antiradical effects of leaf and stem oils of* J. gossypifolia* and reference standard on ABTS radicals; a, b, not significantly different; c, significantly different (*p* < 0.05).

**Figure 3 fig3:**
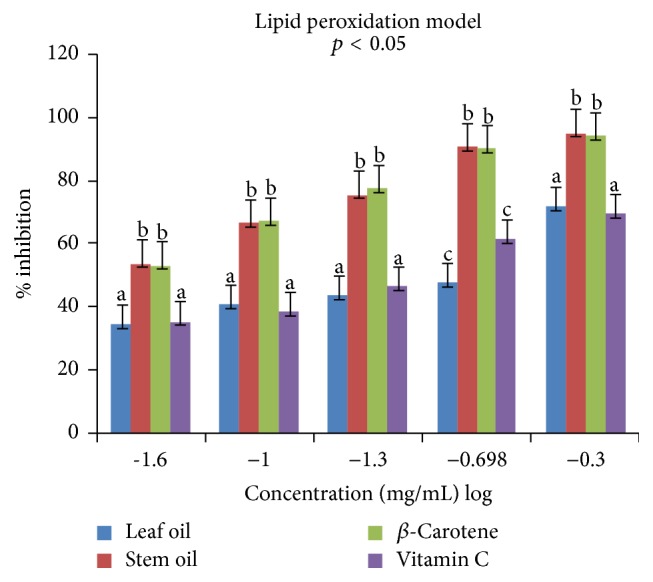
Antiradical effects of leaf and stem oils of* J. gossypifolia* and reference standards on lipid peroxide radicals: a, b, not significantly different; c, significantly different (*p* < 0.05).

**Figure 4:**
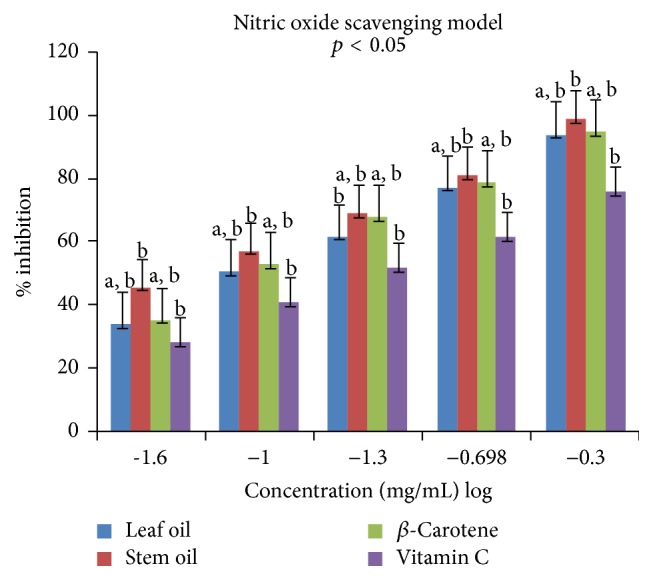
Antiradical effects of leaf and stem oils of* J. gossypifolia* and reference standards on nitric oxide radicals: a, b, not significantly different; b, significantly different (*p* < 0.05).

Antioxidant properties of the leaf and stem oils of* J*.* gossypifolia* were investigated* in vitro* in four different (DPPH, ABTS, LP, and NO) radicals models. The percentage inhibitions of these radicals by the oils and references standards (vitamin C and *β*-carotene) were concentration dependent (0.025 to 0.5 mg/mL) expressed in % inhibition versus log⁡(−1.6  to  −0.3) as presented in Figures 1 [Fig fig2] [Fig fig3]–4. The antiradical effects of LEO and SEO (a, b) on DPPH^•^ were not significantly different at low concentrations (0.025 and 0.05 mg/mL), but at 0.1–0.2 mg/mL, SEO (c) exhibited much higher inhibitory effect than LEO and the reference standards (RS) and effects of LEO and RS were similar (a, b). However at 0.5 mg/mL the SEO displayed similar (a, b) activity as that of the RS (*β*-carotene) while the SEO effect was significantly different (c) from the second RS (vitamin C) as well as the LEO in scavenging DPPH^•^ (Figure 1). The DPPH^•^ antiradical assay is based on the premise that a donor of an atom of hydrogen or an electron is an antioxidant or antiradical and its strength is demonstrated as DPPH^•^ color changes (purple to yellow) in the test sample due to formation of neutral DPPH-H molecule upon absorption of hydrogen from an antioxidant [40]. However, DPPH technique is not a specific radical species test but is for general radicals scavenging potency of an antioxidant [40]. Therefore, to evaluate the precise antiradical efficacy of LEO and SEO of* J*.* gossypifolia*, we quantitatively and qualitatively investigated the presumed antiradical property using two different specific radicals species (LP^•^ and NO^•^) and a cation radical (ABTS^•+^).

Overall, in the four experiments the leaf and stem essential oils of* J. gossypifolia* exhibited effective antiradicals potencies against the different oxidants, indicating they are good electron donors in DPPH and ABTS tests, and displayed strong LP^•^ and valuable NO^•^ antioxidant activity. Assessed by linear regression analysis, the IC_50_ values were calculated while Pearson's correlation analysis and* t*-test were used to test significant difference using SPSS 15.0 for windows (SPSS Inc.). Both oils reduced the DPPH^•^ to a neutral DPPH-H molecule attaining 50% decrease with IC_50_ value of 0.07 ± 0.01 mg/mL for SEO and while that of LEO is 0.32 ± 0.11 mg/mL (Table 4). Significant difference was considered at a level of *p* < 0.05.

The percentages inhibition of the ABTS^•+^ by the SEO and LEO were lower than results obtained in DPPH model, achieving IC_50_ values of 1.34 ± 0.01 and 2.35 ± 0.00 mg/mL, respectively (Table 4). However, unlike in the DPPH assay, the antioxidants completely decolorized the blue color of the oxidant (ABTS^•+^) solutions, turning into neutral molecules (colorless form) from the lowest to highest concentrations (0.025–0.50 mg/mL). This observed effect was stronger with SEO than in LEO, *β*-carotene, and vitamin C. At 0.025 mg/mL the effects of LEO and vitamin C on ABTS^•+^ were comparable (a, b), while SEO (c) exhibited higher effect than RS and LEO (Figure 2). However, as the concentrations increased (0.10–0.20 mg/mL) the antiradical effects of the two reference standards were similar with both lower than SEO (c) but higher than LEO (c). At 0.5 mg/mL SEO demonstrated the highest effect, followed by RS and LEO having the lowest inhibitory effect on ABTS^•^. The discrepancy observed in activities of SEO and LEO against the two different oxidants (DPPH^•^ and ABTS^•+^) could be attributed to many factors including the complexity, polarity, and isomers selectivity of the radicals. In addition, the ease at which the oils solvate the radical's medium may differ and these variables have been reported to influence potency of volatile constituents in inhibiting species of radicals [41].

The lipid peroxide radicals (LP^•^) inhibiting effects of SEO and LEO at different concentrations are showed in Figure 3. The SEO and *β*-carotene exhibited stronger (b, b) antiradical activities than the LEO and vitamin C (a, a) against lipid peroxide induced by ferric sulphate in homogenates of egg yolk. Interestingly, the IC_50_ values of 0.55 ± 0.01 and 0.51 ± 0.00 mg/mL obtained for SEO and *β*-carotene, respectively (Table 4), indicated no significant difference (*p* < 0.05) between volatile oil (SEO) and the reference standard. The antiradical activities of LEO and vitamin C were weak and similar (a, a) at low concentrations (0.025–0.100 mg/mL), with IC_50_ values of 3.31 and 3.01 mg/mL, respectively. However, at 0.2–0.5 mg/mL, their inhibitory activities against lipid peroxide radicals were above average. Notable in the lipid peroxidation model is the significant difference between SEO (b) and vitamin C as well as similar effects of LEO and vitamin C (a, a) in scavenging LP^•^ at 0.025–0.1 mg/mL and 0.5 mg/mL (Figure 3) that may be ascribed to the oils terpenoids, which donate hydrogen atoms to H_2_O_2_, thus reducing it to 2H_2_O.

In the nitric oxide assay, the activities of LEO and SEO to inhibit nitric oxide radical (NO^•^) produced from red-colored complex salt of sodium nitroprusside solution [Na_2_[Fe(CN)_5_NO]·2H_2_O] at different concentrations (0.025–0.5 mg/mL) are showed in Figure 4. The SEO (b) demonstrated stronger inhibitory activity upon NO^•^ compared to LEO as well as the two reference standards at 0.025 and 0.05 mg/mL, while the activities of LEO and vitamin C (RS) are significantly different (a, a) at low concentration (0.025 mg/mL). However, with increasing concentrations (0.1–0.2 mg/mL), SEO and *β*-carotene displayed high and comparable antiradical activity followed by LEO, while vitamin C had the least effect in countering NO^•^ generated (Figure 4). Interestingly at 0.5 mg/mL the LEO and *β*-carotene activities were similar (a, a); however both displayed lower effect than SEO (b). The IC_50_ value obtained for SEO (1.46 ± 0.01 mg/mL) was moderate; however, it was lower than that of LEO (2.10 ± 0.00 mg/mL), carotene (2.03 ± 0.03 mg/mL), and vitamin C (2.91 ± 0.01 mg/mL) as presented in Table 4.

The high phytol content in the EOs in this present study is remarkable and might have enhanced the bioactivity of the oils. Phytol, a diterpenoid alcohol, has been reported by Camilla [42] to demonstrate good antioxidant effect* in vivo* and has high capacity to quench hydroxyl and nitric oxide radicals as well as prevent the formation of lipid peroxides as measured by thiobarbituric acid reactive substances (TBARS). The additive or synergetic effects of identified bioactive constituents in this study (Table 1) may justify the higher bioactivity of the SEO than the LEO. The antibacterial and antioxidant properties of the SEO might have been enhanced by other terpenoids identified even in little amount, for example, menthol (4.87%), *γ*-cadinene (5.49%), and *α*-pinene (5.03%), thus suggesting a possible synergistic interaction between the components [43, 44].

Some recent studies demonstrated that some essential oil compounds that were observed in this present study do possess potent bioactive properties [10, 12, 18, 45]. Menthol, for example, which was found in SEO, has been reported to demonstrate very high antimicrobial, antioxidant, and anti-inflammatory activities [46]. Furthermore, limonene has been proven in previous studies [47] as a strong bioactive monoterpene and its proapoptotic effects on human gastric cancer and its antitumor and antimetastasis activities have been demonstrated. Takahashi [48] reported that terpinene, another monoterpene hydrocarbon also identified in the SEO of* J. gossypifolia*, has the ability to inhibit low density lipoprotein oxidation even in the formation phase. In addition, the main component phytol, which was identified in the two oils, could have possibly reacted with DPPH^•^, ABTS^•+^, LP^•^, and NO^•^ through various mechanisms suggested by Foti and Amorati [49]. The result in this current study is in agreement with other reports that have implicated aliphatic terpene with antiradical properties, while effect of hydrocarbon monoterpene which is cyclic with double bonds is similar to the property of phenolic compounds or *α*-tocopherol [5, 6, 10, 17]. Activity of SEO against* E. coli*,* E. faecium*, and* S. aureus* as well as scavenging different radicals as observed in this present study is quite noteworthy. These observations may therefore suggest that SEO of* J. gossypifolia* could possibly be a new potent candidate in the search for lead compounds for the management of infectious and oxidative stress-related disorders such as Alzheimer's disease (AD), cancers, diabetic nephropathy, and arteriosclerosis [50–52].

## 4. Conclusion

This present study indicates that, apart from the local uses of the leaf and stem of* J. gossypifolia*, the essential oil contained strong bioactive phytochemicals and they are good prospect as new antimicrobial agent and an alternative to synthetic antioxidant and could be used as food preservatives on further investigation.

## Supplementary Material

Figure 5: Total ion chromatogram of the leaves essential oil of *J. gossypifolia*.
Figure 6: Total ion chromatogram of the stem essential oil of *J. gossypifolia*.

## Figures and Tables

**Table 1:** Essential oils constituents in the leaf and stem of *Jatropha gossypifolia*.

Constituent^a^	KI^b^	Composition (%)		Methods of identification	MS data^c^	Q A^d^
		Leaf	Stem			
*α*-Pinene	927	—	5.03	KI, MSD	93, 79, 41, 136	97
*β*-Pinene	938	4.60	t	KI, MSD	93, 69, 41, 136	90
Camphene	954	—	2.79	KI, MSD	93, 69, 41, 136	98
*α*-Terpinene	1016	—	10.61	KI, MSD	93, 77, 136, 121	97
Limonene	1028	—	12.42	KI, MSD	68, 93, 107, 121	98
Linalool	1091	9.81	—	KI, MSD	71, 43, 69, 55	95
Menthol	1142	t	4.87	KI, MSD	79, 43, 41, 105	90
*β*-Bisabolene	1384	0.30	0.58	KI, MSD	204, 69, 41, 43	90
*α*-Aromadendrene	1386	3.65	10.48	KI, MSD	159, 91, 41, 220	90
*α*-Cadinene	1460	—	1.83	KI, MSD	161, 43, 105, 204	96
Germacrene D	1464	—	12.30	KI, MSD	20, 161, 32, 105	98
Farnesene	1471	2.60	0.27	KI, MSD	69, 93, 107, 133	96
*γ*-Cadinene	1486	4.21	5.49	KI, MSD	161, 43, 105, 204	97
*α*-Muurolene	1499	—	0.48	KI, MSD	121, 95, 43, 105	95
Viridiflorol	1530	8.27	—	KI, MSD	32, 79, 55, 109	90
Pentadecen-5-yne	1538	9.43	—	KI, MSD	79, 67, 41, 109	96
Bisabolol	1534	1.40	—	KI, MSD	39, 204, 69, 41	90
Germacrene B	1559	0.73	0.13	KI, MSD	120, 161, 32, 105	95
*α*-Copaene	1634	—	12.20	KI, MSD	105, 119, 161, 141	95
Dodecanoic acid	1975	3.23	—	KI, MSD	29, 60, 73, 129	98
Hexadecanoic acid	1968	5.06	—	KI, MSD	60, 73, 43, 256	97
Octadecanal	1999	8.60	t	KI, MSD	41, 57, 82, 96	90
Phytol	2045	33.40	18.05	KI, MSD	71, 57, 41, 123	90
9,17-Octadecadienal	2112	0.21	t	KI, MSD	280, 265, 279, 73	90

Total oil content (%)		**98.70**	**97.50**			
Yield of oil		**0.32**	**0.21**			

^a^Constituent elution order in column HB-5; ^b^Kovat's index, ^c^some of the *m/z* for most abundant peaks in the mass spectrum, ^d^percentage of GC/MS library quality assurance of constituent in SEO/LEO, MSD = mass spectra data; RI = retention index relative to C_9_–C_23_ on the column HB-5, t = less than 0.05%.

**Table 2:** Minimum inhibitory concentration (MIC) values (mg/mL) for essential oils of *J. gossypifolia* against bacteria strains.

Bacteria	Leaves oil	Stem oil	Ciprofloxacin Positive control	DMSO Negative control
*Staphylococcus aureus *	0.10 ± 0.01 NG	0.05 ± 0.01 NG	0.05 ± 0.01 NG	0.5 mL VG
*Enterococcus faecium*	0.05 ± 0.00 NG	0.025 ± 0.00 NG	0.05 ± 0.02 NG	0.5 mL VG
*Escherichia coli*	0.10 ± 0.01 NG	0.05 ± 0.00 NG	0.05 ± 0.01 NG	0.5 mL VG

Significant difference was considered at a level of *p* < 0.05; NG: no growth; VG: visible growth.

**Table 3:** Minimum bactericidal concentration (MBC) values (mg/mL) for essential oils of *J. gossypifolia* against bacteria strains.

Bacteria	Leaves oil	Stem oil	Ciprofloxacin Positive control	DMSO Negative control
*Staphylococcus aureus *	Bacteriostatic at 0.10 ± 0.01 VG	Bactericidal at 0.05 ± 0.01 NG	Bactericidal at 0.05 ± 0.01 NG	0.5 mL VG
*Enterococcus faecium*	Bacteriostatic at 0.05 ± 0.03 NG	Bactericidal at 0.025 ± 0.00 NG	Bactericidal at 0.05 ± 0.02 NG	0.5 mL VG
*Escherichia coli*	Bacteriostatic at 0.10 ± 0.01 VG	Bactericidal at 0.10 ± 0.00 NG	Bactericidal at 0.05 ± 0.03 NG	0.5 mL VG

Significant difference was considered at a level of *p* < 0.05; NG: no growth; VG: visible growth.

**Table 4:** Antiradical capacity of essential oils extracted from *J. gossypifolia *[IC_50_ (mg/mL)].

Activity	*J. gossypifolia*		Reference compounds	
	Leaf oil	Stem oil	Vitamin C	*β*-Carotene
DPPH^•^	0.32 ± 0.11	0.07 ± 0.01	1.64 ± 0.01	1.50 ± 0.12
ABTS^•+^	2.35 ± 0.00	1.34 ± 0.10	2.43 ± 0.12	2.06 ± 0.11
LP^•^	3.31 ± 0.04	0.55 ± 0.01	3.00 ± 0.01	0.51 ± 0.00
NO^•^	2.10 ± 0.00	1.46 ± 0.01	2.91 ± 0.01	2.03 ± 0.10

The IC_50_ (mg/mL) was obtained from standard curve for each oil and reference drugs. The lower the IC_50_, the higher the antiradical strength. Significant difference was considered at a level of *p* < 0.05. Values are mean ± SD, *n* = 3.
