# Supplementary material for: Antibacterial and Antioxidant Properties of the Leaves and Stem Essential Oils of Jatropha gossypifolia L
Source: Biomed Res Int. 2016 Oct 24;2016:9392716. doi: 10.1155/2016/9392716 (PMC5098104; doi:10.1155/2016/9392716)
Supplement: Supplementary file 1 — Figure 5: Total ion chromatogram of the leaves essential oil of J. gossypifolia. Figure 6: Total ion chromatogram of the stem essential oil of J. gossypifolia. [file 9392716.f1.pdf]

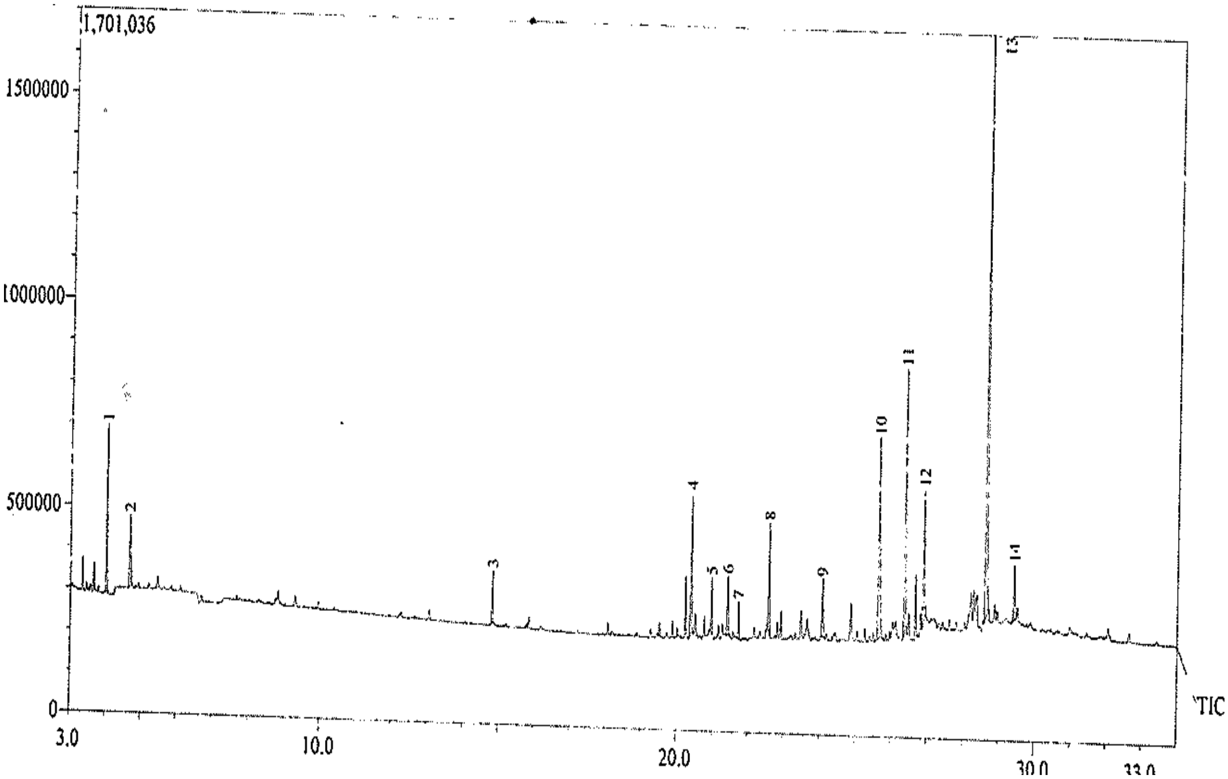

Figure 5: Total ion chromatogram of the leaves essential oil of *J. gossypifolia*

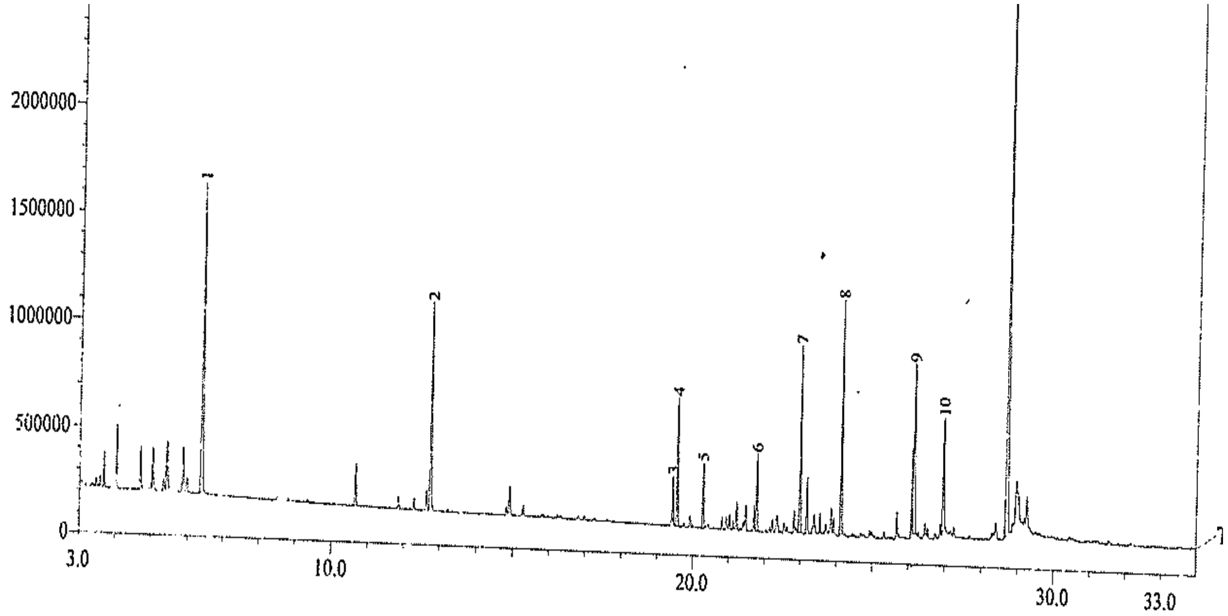

Figure 6: Total ion chromatogram of the stem essential oil of *J. gossypifolia*
